# Enzymatic Debridement for Burn Wound Care: Interrater Reliability and Impact of Experience in Post-intervention Therapy Decision

**DOI:** 10.1093/jbcr/iraa218

**Published:** 2020-12-30

**Authors:** Laura C Siegwart, Arne H Böcker, Yannick F Diehm, Dimitra Kotsougiani-Fischer, Stella Erdmann, Benjamin Ziegler, Ulrich Kneser, Christoph Hirche, Sebastian Fischer

**Affiliations:** 1 BG Trauma Center Ludwigshafen, Department of Hand, Plastic and Reconstructive Surgery, Microsurgery, Burn Center, Hand and Plastic Surgery, University of Heidelberg, Germany; 2 Institute for Medical Biometry and Informatics, University of Heidelberg, Germany

## Abstract

Enzymatic debridement (ED) has become a reliable tool for eschar removal. Although ED application is simple, wound bed evaluation and therapy decision post-intervention are prone to subjectivity and failure. Experience in ED might be the key, but this has not been proven yet. The aim of this study was to assess interrater reliability (IR) in post-intervention wound bed evaluation and therapy decision as well as the impact of experience. In addition, the authors introduce video assessment as a valuable tool for post-ED decision-making and education. A video-based survey was conducted among physicians with various experiences in ED. The survey involved multiple-choice and 5-point Likert scale questions about professional status, experience in ED, confidence in post-ED wound bed evaluation, and therapy decision. Subsequently, videos of 15 mixed pattern to full-thickness burns immediately after removal of the enzyme complex were demonstrated. Participants were asked for evaluation of each burn wound, including bleeding pattern and consequent therapy decision. IR ≥ 80% was considered as a consensus. Responses were stratified according to participants’ experience in applying ED (<10, 10–19, 20–49, and ≥50 applications). IR was assessed by chi-square test (raw agreement [RA]; ≥80% was considered as a consensus) and by calculation of Krippendorff’s alpha. In addition, expert consensus for therapy decision was compared with the actual clinical course of each shown patient. Last, participants were asked for their opinion on video as an assessment tool for post-ED wound bed evaluation, decision-making, and training. Thirty-one physicians from 11 burn centers participated in the survey. The overall consensus (raw agreement [RA] ≥ 80%) in post-ED wound bed evaluation and therapy decision was achieved in 20 and 40%, respectively. Krippendorff’s alpha is given by 0.32 (95% confidence interval: 0.15, 0.49) and 0.31 (95% confidence interval: 0.16, 0.47), respectively. Subgroup analysis revealed that physicians with high experience in ED achieved significantly more consensus in post-intervention wound bed evaluation and therapy decision compared with physicians with moderate experience (60 vs 13.3%; *P* = .02 and 86.7 vs 33.3%; *P* = .04, respectively). Video analysis was considered a feasible (90.3%) and beneficial (93.5%) tool for post-intervention wound bed evaluation and therapy decision as well as useful for training purposes (100%). Reliability of wound bed evaluation and therapy decision after ED depends on the experience of the rating physician. Video analysis is deemed to be a valuable tool for ED evaluation, decision-making, and user training.

First introduced by Rosenberg et al^1^ in 2004, enzymatic eschar removal has become a valuable alternative to surgical excision in mixed pattern, deep partial-, and full-thickness burns in European burn centers. The bromelain-based enzymatic complex (NexoBrid^TM^, MediWound, Rüsselsheim, Germany) is a reliable debridement tool, which has the unique capacity to preserve healthy and viable dermis and thus removes burned tissue selectively. In detail, the active enzyme complex involves several collagenases that selectively break down thermally damaged collagen structures, which will subsequently be removed by wound irrigation. Collagen is the main component of the skin and underlying tissues. Therefore, by removing thermally damaged collagen, a sufficient burn wound debridement can be achieved. This can accelerate the healing of the burn wound and reduces the extent of subsequent skin grafting if required.^2^ Of note, the enzymatic complex comes in the form of an ointment that is applied topically on the burn wound, and if burn wound location allows for regional anesthesia, bedside application is feasible. Therefore, enzymatic debridement (ED) is easily applicable and readily available without using valuable operating room capacity. The application of ED is particularly advantageous for severe burns of hands^3^ to prevent burn-induced compartment syndrome and invasive surgical escharotomy in upper extremity burns by prompt application^4^ as well as for further indications such as genital and facial burns.^5, 6^ Its role and implication for burn centers in Europe have been methodologically addressed in two consensus meetings in 2017 and 2019.^7, 8^

However, ED comes with several limitations. One of the biggest challenges, yet crucial for successful clinical application, is the interpretation of the wound bed after debridement. Clinical criteria such as the structure and color of the wound bed and, in particular, the pattern and dynamics of bleedings allow for conclusions about the burn depth.^9^ The bigger the diameter of the bleeding points and the larger the interspace, the deeper is the dermis affected. While in deep burns, ED should soon be followed by autografting, and superficial burns can be left for conservative treatment.^8^ Of note, clinical evaluation of the wound bed and associated decision for surgical or conservative therapy are only feasible for a short time after ED, because subsequently a so-called pseudo-eschar consisting of fibrin and other exudates is covering the wound and makes assessment or reevaluation impossible.

Furthermore, drawing conclusions from color of the wound bed, diameter, or interspace of bleeding points is quite subjective and based on personal experience.

This can make the decision for post-intervention care susceptible to error with subsequent therapy failure.

The aim of this study was to evaluate the impact of personal experience on wound bed evaluation and subsequent therapy planning after ED of burn wounds. In addition, the authors introduce video as a valuable tool for post-ED wound bed evaluation and decision-making as well as for education purposes.

## MATERIALS AND METHODS

**Figure 1.**
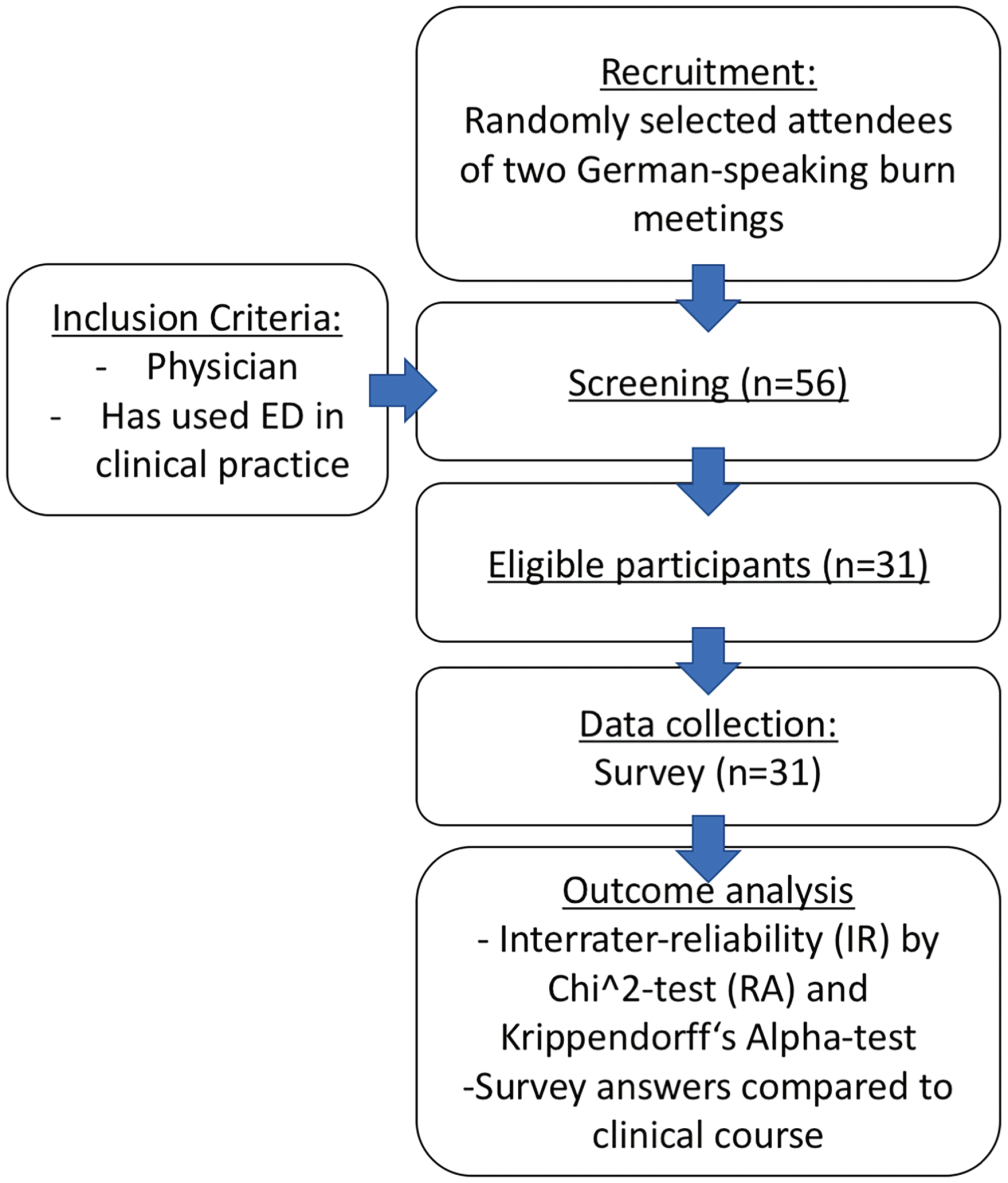
Diagram of study design.

A survey was conducted from December 2019 to January 2020. Attendees of the 3rd German-speaking expert meeting for ED (December 12, 2019, Kelkheim, Germany) as well as the 38th annual meeting of the German-speaking Society for burn medicine (DAV, January 15–18, 2020, Zell am See, Austria) were randomly selected and asked to participate in a survey about clinical utilization of ED. Inclusion criteria were being a physician and to have utilized ED in clinical practice at least once. The survey was conducted anonymously, ie, without any personal information about the participant. The study was in accordance with the local ethics committee and the declaration of Helsinki. The study design is depicted in Figure 1.

The survey was subdivided into parts A, B, and C. In part A, the professional status (AQ1), country of origin (AQ2), and experience with ED (AQ3) were asked by means of multiple-choice questions. Confidence in ED application for burn wound care (AQ4) and confidence in post-ED wound bed evaluation and therapy decision (AQ5) were surveyed using 5-point Likert scale questions.

In part B, 15 video sequences (mean length 12.6 s, range 7–19 s) of burn wounds after ED were demonstrated, and participants were asked for wound bed evaluation including bleeding pattern and consequent therapy recommendation (BQ1–15a and b). Videos (4K, 60 fps) were recorded with a commercially available smartphone (Apple iPhone 11 Pro, Apple Inc., Cupertino, California) from September 1, 2019, to November 30, 2019, and included superficial partial, mixed pattern, deep partial-, or full-thickness burn wounds at the upper extremity that underwent ED (NexoBrid^TM^) at our department. ED was applied according to an established in-house protocol.^4^ Videos contained a full-size overview as well as a zoom-in sequence of the wound bed immediately after removal of the enzyme complex and debris (Video 1 and Supplementary Videos 2–15). Videos did not reveal any patient identifying information. During the survey, videos were demonstrated in a separate room on a high-resolution retina display 15.4″ (Apple Mac Book Pro, Apple Inc.) and light conditions were equal between each demonstration. Wound bed evaluation (BQ1–15a) and therapy recommendation (BQ1–15b) were surveyed using multiple-choice questions. Answer choices for wound bed evaluation (uniform red or pink wound bed [2a°]/pinpoint punctate bleeding [2a°]/large diameter bleeding points [2b°]/exposed fat or functional structures [3°]/other character) were based on the algorithm previously published by Hirche et al.^8^

**Video 1.**
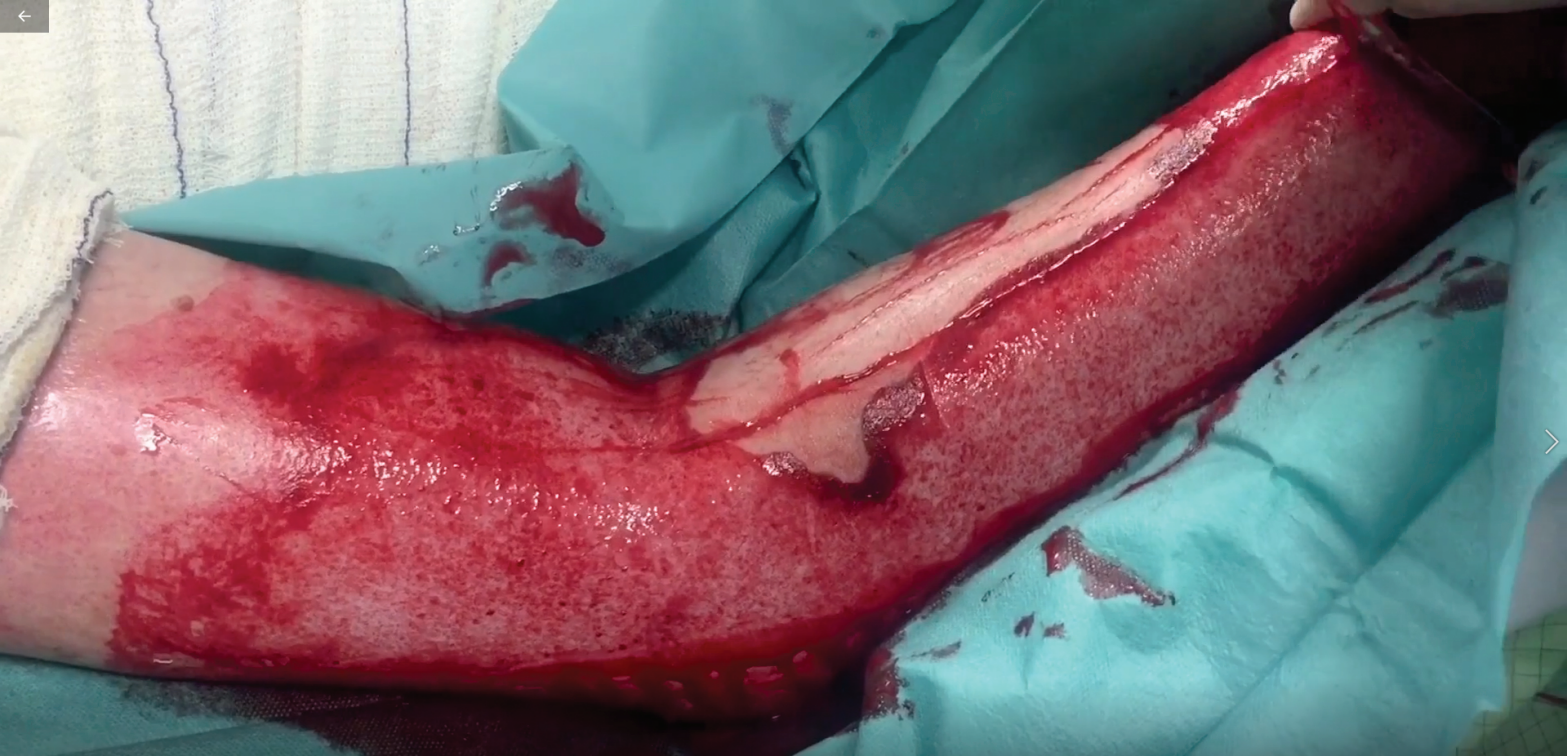
Example video sequence of wound bed directly after ED removal (video of question BQ7a/b); 100% of ED expert users (≥50 ED applications) voted for large diameter bleeding points (BQ7a) and 80% recommended an operative therapy post-ED (BQ7b).

In part C, 5-point Likert scale questions were used to rate feasibility (CQ1) and benefit (CQ2) of video as an “assessment tool” after ED. Last, participants were asked to evaluate post-intervention video to educate ED users (CQ3).

Questions and possible answers of the entire survey are provided in Table 1.

**Table 1.** Survey as handed out to the participants. Questions BQ1–15a and b were asked after demonstration of each video (Video 1 and Supplementary Videos 2–15, respectively)

Video-based wound bed evaluation and therapy decision after enzymatic debridement (ED) for burn wound care
Please select one choice per question in parts A, B, and C of the survey.
Video-independent survey in parts A and C.
Video-dependent survey in part B.
Part A:
**AQ1: What is your professional status?**
chief physician/ consultant physician/ senior resident
**AQ2: In which country is your burn center located?**
Germany/ Switzerland/ Austria
**AQ3: How often have you used ED for burn wound care?**
<10 applications/ 10–19 applications/ 20–49 applications / ≥50 applications
**AQ4: I feel confident in ED application for burn wound care.**
Strong agreement/ agreement/ neutral/ disagreement/ strong disagreement
**AQ5: I feel confident in wound bed evaluation and therapy decision post-ED.**
Strong agreement/ agreement/ neutral/ disagreement/ strong disagreement
Part B:
**BQ1–15a: How do you evaluate the wound bed post-ED (video 1–15)?**
Uniform red or pink wound bed/ pinpoint bleedings/ large diameter bleeding points/ exposed fat or functional structures/ other character
**BQ1–15b: What therapy would you initiate (video 1–15)?**
Conservative care/ surgical therapy (eg, skin grafting)
Part C:
**CQ1: Video is a feasible tool for post-ED wound bed evaluation and therapy decision.**
Strong agreement/ agreement/ neutral/ disagreement/ strong disagreement
**CQ2: Video is a beneficial tool for post-ED wound bed evaluation and therapy decision.**
Strong agreement/ agreement/ neutral/ disagreement/ strong disagreement
**CQ3: Video is useful for training users in post-ED wound bed evaluation and therapy decision.**
Strong agreement/ agreement/ neutral/ disagreement/ strong disagreement

### Interrater Reliability and Statistics

Interrater reliability (IR) was calculated for all participants (*n* = 31) as well as for subgroups according to participants’ experience with ED (<10 applications, 10–19 applications, 20–49 applications, and ≥50 applications). IR was assessed by chi-square test (raw agreement [RA]) and by calculation of Krippendorff’s alpha with associated 95% confidence intervals (CIs).^10–12^ Krippendorff’s alpha is known as conservative measures of agreement for categorical data, for which there are generally no binding limit values for interpretation; however, it may be interpreted like Cohen’s kappa.^13–15^ RA of ≥80% was considered as a consensus. This means, that a consensus was reached, if the same answer in the survey was chosen by 80% or more of the participants. Prism 8.3.0 software (GraphPad Software, San Diego, California) was used for chi-square test, and significance was set at *P* < .05. For Krippendorff’s alpha, the statistical software R, in particular the R package irrCAC, was used.^16, 17^

Finally, the actual clinical course of each patient shown in Video 1 and Supplementary Videos 2–15 was reviewed in the medical records (conservative or surgical therapy) and compared with the answers given by ED expert users (≥50 applications) in questions BQ1–15b.

## RESULTS

**Figure 2.**
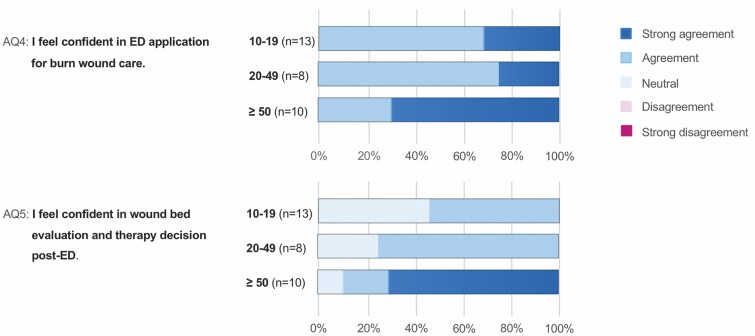
Video survey part A: responses to 5-point Likert scale questions AQ4 and AQ5 (left) are presented as color coded as a percentage of physicians with moderate experience (10–19 applications), of physicians with advanced experience (20–49 applications), and of physicians with great experience (≥50 applications) (central). Color code of choices (right).

In total, 31 physicians (9 chief physicians, 12 consultant physicians, and 10 senior residents) from 11 burn centers (9 in Germany, 1 in Austria, and 1 in Switzerland) participated in the survey. All questionnaires were answered completely and could be included in the study. A total of 13 participants had moderate experience (10–19 applications), 8 had advanced experience (20–49 applications), and 10 had high experience (≥50 applications) in ED for burn wound care. All participants stated to feel confident in ED for burn wound care (AQ4). About 70% (7/10) of physicians with great experience strongly agreed to feel confident in post-intervention wound bed evaluation and decision-making, while 46% (6/13) of physicians with moderate experience choose the neutral answer (AQ5). The detailed responses to 5-point Likert scale questions AQ4 and AQ5 are depicted in Figure 2.

**Figure 3.**
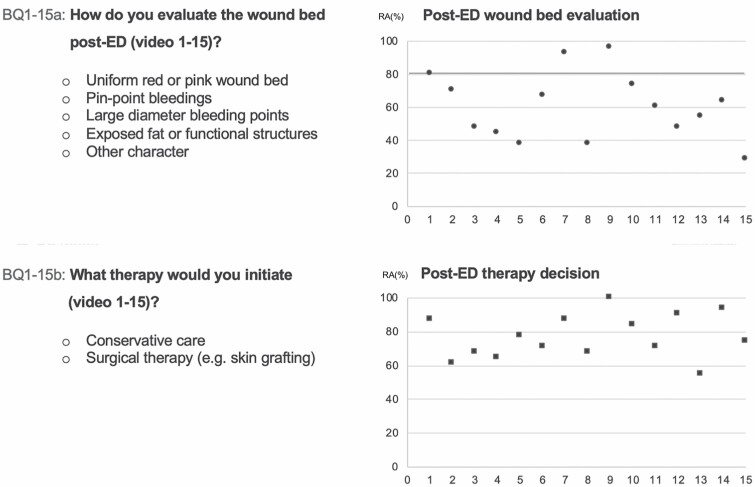
Survey questions and multiple-choice answer possibilities of questions BQ1–15a (upper row, left) and BQ1–15b (lower row, left). Diagram of raw agreement (RA) of all participants’ responses to each multiple-choice questions BQ1–15a (right upper diagram) and BQ1–15b (right lower diagram). The x-axis of diagrams shows questions BQ1–15a (upper diagram) or BQ1–15b (lower diagram), and the y-axis shows RA in percent. For example, BQ1a achieved a RA of 80%. This means that 80% of participants chose the same answer.

In part B of the survey, the overall consensus (all participants [*n* = 31]; RA ≥ 80%) in wound bed evaluation and in treatment recommendation was achieved in 20% (3/15) and in 40% (6/15) of the demonstrated burn wounds, respectively. The RA of all participants’ responses to questions BQ1–15a and BQ1–15b is shown in Figure 3. Krippendorff’s alpha and associated 95% CI are given by 0.32 (0.15, 0.49) and 0.31 (0.16, 0.47), respectively.

With respect to subgroups, consensus (RA ≥ 80%) in wound bed evaluation was achieved in 60% (9/15), 33.3% (5/15), and 13.3% (2/15) of demonstrated videos in physicians with great experience (*n* = 10), advanced experienced (*n* = 8), and moderate experience (*n* = 13) with ED, respectively. Krippendorff’s alpha and associated 95% CI are given by 0.5 (0.29, 0.71), 0.35 (0.15, 0.55), and 0.25 (0.07, 0.42), respectively.

Consensus (RA ≥ 80%) in therapy recommendation was achieved in 86.7% (13/15), 60% (9/15), and 33.3% (5/15) of demonstrated videos in subgroups of physicians with high experience (*n* = 10), advanced experienced (*n* = 8), and moderate experience (*n* = 13) with ED, respectively. Krippendorffs alpha and associated 95% CI are given by 0.51 (0.34, 0.68), 0.42 (0.21, 0.63), and 0.2 (0.04, 0.35), respectively.

**Figure 4.**
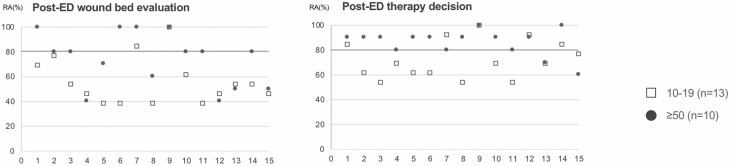
Subgroup analysis of survey questions BQ1–15a (left) and BQ1–15b (right). The x-axis shows questions BQ1–15a (left diagram) and BQ1–15b (right diagram), and the y-axis shows raw agreement (RA) in percent. Subgroup with moderate experience in ED application (10–19 applications) and subgroup with high experience (≥50 applications) are depicted in squares and points, respectively. For example, question BQ1a revealed a raw agreement of 70% and 100% in the moderate and high experience groups, respectively. This means that 70% and 100% of participants with moderate and high experience, respectively, chose the same answer.

The consensus in wound bed evaluation and therapy recommendation was significantly lower in the moderate experience subgroup compared with the high experience subgroup (*P* = .02 and *P* = .04, respectively). Comparison of RAs is shown in Figure 4.

Comparison between expert consensus about therapy decision (BQ1–15b) and the actual clinical course of each patient revealed a concordance of 66.6% of cases (10/15; Table 2).

**Table 2.** Medical record review of each patient shown in video 1 and suppl. Video 2–15, including age, gender, burn mechanism, TBSA, TBSA and extremity treated with enzymatic debridement (ED), burn depth, post-ED therapy, and accordance with experts’ consensus in therapy decision (BQ1–15b) achieved in the survey

				ED Application				Post-ED Therapy		
Video	Age.Gender	Burn Mechanism	TBSA (%)	Day	TBSA (%)	Location	Burn Depth	Conservative: Reepithealization (day)	Operative: Surgery (day)	Accordance with Experts’ Consensus in Therapy Decision (BQ1–15b)
Suppl. Video 2	25.M	Scald by hot fat	9	0	4	Right lower arm	2b	–	6	No
Suppl. Video 3	16.M	Gas explosion	19	0	8	Left hand	2b	–	5	Yes
Suppl. Video 4	41.M	Scald by hot fat	31	0	6	Right hand	2a	18	–	No
Suppl. Video 5	22.M	Scald by hot fat	50	0	12	Right lower arm	2b	–	2	Yes
Suppl. Video 6	43.M	Flash fire	22	0	4	Right lower arm	2a	14	–	Yes
Suppl. Video 7	16.M	Gas explosion	19	0	8	Left hand	2a	10	–	Yes
Suppl. Video 1	59.M	Electric arc	65	0	5	Right upper arm	2b	–	7	Yes
Suppl. Video 8	48.F	Flash fire	17	0	9	Left hand	2a	10	–	Yes
Suppl. Video 9	91.F	Scald by hot water	18	2	10	Left lower arm	3	–	7	Yes
Suppl. Video 10	25.M	Scald by hot fat	9	0	4	Right hand	2b	–	6	No
Suppl. Video 11	59.M	Electric arc	65	0	5	Right hand	2b	–	7	No
Suppl. Video 12	22.M	Scald by hot fat	50	0	12	Left hand	2b	–	2	Yes
Suppl. Video 13	36.M	Fire flame	16	0	6	Left lower arm	2a	14	–	No
Suppl. Video 14	91.F	Scald by hot water	18	2	10	Left upper leg	3	–	7	Yes
Suppl. Video 15	22.M	Deflagration	14	0	5	Right hand	2b	–	5	Yes

B1–B15: patients’ clinical course and retrospective burn depth assessment.

**Figure 5.**
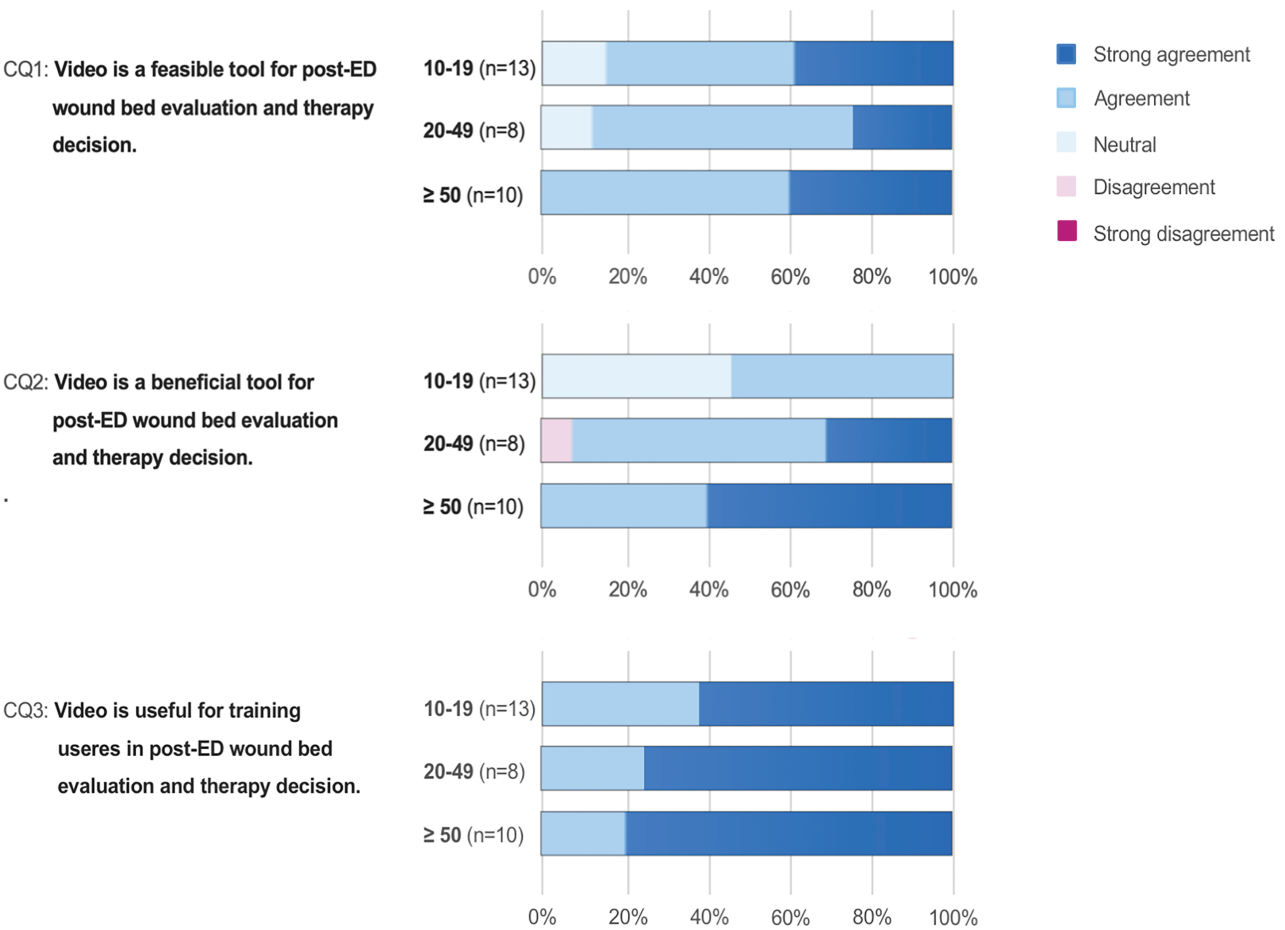
Video survey part C: responses to 5-point Likert scale questions CQ1, CQ2, and CQ3 (left) are presented as color coded in percentage of physicians with moderate experience (10–19 applications), of physicians with advanced experience (20–49 applications), and of physicians with high experience (≥50 applications) (central). Color code of choices (right).

In part C of the survey, 90.3% (28/31) of the participants stated video to be a feasible tool and 93.5% (29/31) stated video to be a beneficial tool for wound bed evaluation and therapy decision post-ED. All participants (31/31) stated video to be a useful tool for education of users in post-ED wound bed evaluation and decision-making. The detailed responses to 5-point Likert scale questions CQ1, CQ2, and CQ3 are shown in Figure 5.

## DISCUSSION

**Figure 6.**
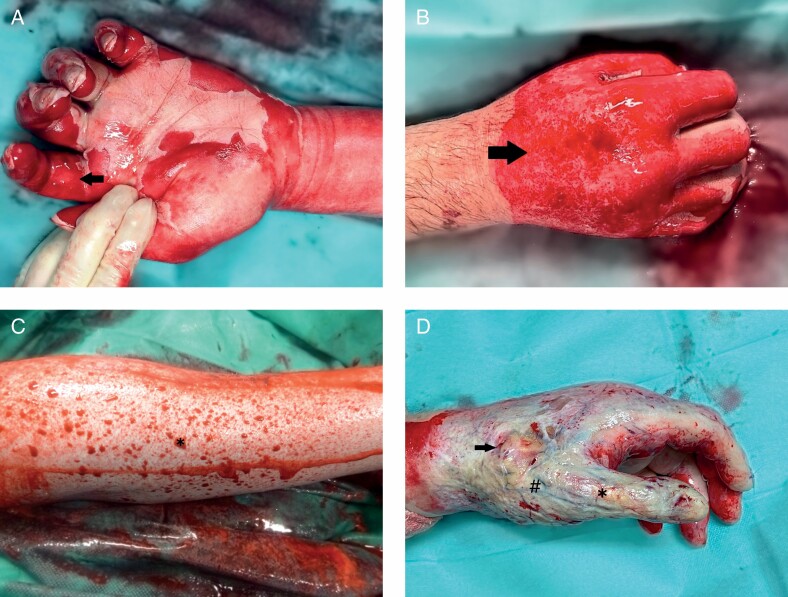
Photographic image of enzymatically debrided burns subsequent to removal of the necrotic debris and inactivated enzyme complex: A. Superficial partial-thickness burn: uniform pink and red shaded wound bed (arrow) with abundance of small diameter pinpoint bleeders. B. Intermediate partial-thickness burn: irregular shaded pink and red wound bed with numerous pinpoint bleeders (arrows). C. Deep partial-thickness burn: few large-diameter bleeding points (asterisk) on pale wound bed, which is depressed in relation to the surrounding healthy skin. D. Full-thickness burn: exposed subcutaneous fat (arrow), vessels (hashtag), and functional structures (asterisk).

ED is an effective tool for eschar removal in partial-thickness to full-thickness burns with increasing popularity in European burn centers. Feasibility, safety, and efficacy of ED have been validated in numerous studies.^2, 3, 5, 18, 19^ However, promptly after ED, the result must be interpreted thoroughly, and, subsequently, further conservative or surgical therapy initiated. Especially in deep partial- and full-thickness burns, the decision for skin grafting or other reconstructive procedures is paramount for successful clinical outcome.^8^ Therefore, evaluation of the wound shortly after the removal of the inactivated enzyme complex and eschar remnants has evolved into not only the most critical but also demanding point for successful ED application. The present study revealed high variability in the decision for conservative care or surgical therapy post-ED—a consensus (≥80% IR) was achieved in merely 40%. Decision-making is based on clinical evaluation of the wound bed color, morphology, as well as pattern and dynamics of bleeding points. In 2018, the first algorithm for post-ED wound bed evaluation and therapy decision was outlined in an European consensus^7^: A uniform pink or red wound base (Figure 6A) and pinpoint bleeding pattern (Figure 6B) indicate good chances for spontaneous healing, while scattered big red circles on pale wound base (Figure 6C) indicate reduced healing potential with deep dermal affection and skin grafting should be considered. Exposed subdermal fat or functional structures (Figure 6D) indicate complete loss of viable dermis, and, in those cases, reconstructive procedures should be performed. For ED users, this algorithm provides valuable features for outcome prognosis and therapy decision based on burn experts’ mutual experience. Even so, the interpretation of criteria such as pinpoint bleeding or large diameter red circles is highly subjective and considerably influenced by personal experience in applying ED. Our results substantiate this hypothesis: Physicians with high experience in ED (≥50 applications) achieved significantly more consensus in post-intervention wound bed evaluation (60 vs 13.3%, *P* = .02) and therapy decision (86.7 vs 33.3%, *P* = .04) compared with physicians with moderate experience (10–19 applications). Similarly, conventional burn depth evaluation, which relies on the subjective evaluation of external features such as wound appearance, capillary refill, and burn wound sensibility to touch and pinprick, is accurate in 60 to 75%, even when conducted by an experienced burn surgeon.^20^

However, considering the impact of experience in applying ED, for the inexperienced or moderate experienced ED user, it might be recommendable to get expert advice on-site or via telemedicine to provide the advantages of the minimally invasive method to patients and experience successful clinical outcome. In this context, the European consensus guidelines for ED in burns summarized^7^:

Photography … is strongly recommended to shorten the learning curve for the whole burn team, and to have the chance to discuss wound evaluation and specific issues on ED and patterns.

The so-called “remote expert consultation” using digital media is well trialed in acute care of burns due to the superficial and visual nature and a limited number of burn centers, particularly in low-income countries.^21^ Roa et al compared clinical evaluation of burn depth with the photographic image and found similar success of both methods in 90%.^22^ With the upcoming use of mobile phones, Shokrollahi et al suggested to communicate images for remote evaluation of TBSA and burn depth and found a high correlation between remote and live evaluation,^23^ which was confirmed by Saffle et al.^24^ In this context‚ remote evaluation of burn wounds by experienced physicians has been shown to be more precise and to correlate more closely with live evaluation compared with estimates by less experienced healthcare professionals at the point of care. Nowadays telemedicine has been implemented in burn medicine and provides access to high-quality medical service in the diagnosis of TBSA and burn depth, triage, and transfer decision, as well as therapy decision based on expert’s advice centralized within a limited number of specialized burn centers to patients in remote geographic distribution.^25–27^ The electronic exchange of interactive or store and forward video or imaging decreases medical errors in diagnosis and treatment and improves inexperienced physicians’ experience and skills by the exchange to a specialist.^21^ However, in many countries, privacy, legal aspects, and limited resources complicate the implementation and expansion of this powerful access to specialized health care.

In applying ED, the authors noticed the advantage brought by the easy and inexpensive availability of video using mobile phones equipped with a digital camera, the technology for which is becoming increasingly sophisticated. Since January 2019, the authors started to record the burn wound after removal of the dissolved necrotic dermis and the residues of the enzyme complex by scraping. In addition to moderate or advanced users’ on-site wound bed evaluation, an in-house expert consultation was conducted within 24 hours, including the hole burn team to verify the therapy decision, ensure the quality of care, and refine users’ learning curve. In contrast to the photographic image, high-quality video visualizes the debrided wound in detail, particularly the dynamics and intensity of post-intervention bleeding and its specific pattern. To be complete, it should be stated that even high-quality video limits three-dimensionality and exists outside of the context of tactile examination. However, since visual features are paramount, to the authors’ opinion, video analysis is a feasible tool for wound bed evaluation and therapy decision, which was similarly confirmed by expert users in this study—and expected to be superior to photography.

In addition, Nelson et al^27^ showed that the use of photographs improved patient care for review of healing or complications in burn therapy. Also, Fischer et al^28^ demonstrated video analysis to negotiate inaccuracy and subjectivity in outcome analysis of facial movements and functions after face transplant. Likewise, video allows for transparency in post-ED decision-making, which might be the first step to ensure the quality of care using ED, a quite new method in burn wound care. With these aspects in mind, video analysis is a beneficial tool when using ED—a view that has been shared by experienced users in this study. Moreover, all physicians included in this study supported video for training ED users irrespective of their level of experience, which might likewise reflect and fill up the current lack of visual references available to date. For this reason, the authors are currently preparing a video tutorial to provide visual education and objective post-intervention therapy algorithms for all ED users. In the future, video-based telemedicine might be the key for successful implementation and application of ED in burn wound care in countries such as the United States to share the expertise and shorten the learning curve.

There are several limitations to this study. First, assessment of the wound bed after ED with means of a video sequence is not an established modality. The current golden standard for post-ED wound bed assessment is live evaluation. However, this would prohibit the inclusion of various physicians of multiple centers and thus not allow for comparison of different experience levels with ED on evaluation of the post-ED wound bed. Nevertheless, a strong indicator that video-based assessment comes close to live evaluation was the concordance between survey results of experienced ED users and live evaluation performed in our center at the time when the video was taken. The latter was also utilized as a reference for statistical analysis. Secondly, the experience of each survey participant could not be verified by any data and was only based on the information provided by the participant himself. However, since the survey was undertaken anonymously without any further personal information about the participant, we do not believe that false information about experience level with ED would have led to any benefits for the participant.

With these limitations in mind, the authors present a unique study involving randomly selected ED users from various burn trauma facilities to shed light on the challenge of wound bed evaluation after ED. Of note, ED is readily available and easily applicable and thus has the potential to become a game changer in burn wound care. Although several burn trauma facilities utilize ED in clinical practice, outcomes of various successes are reported, which is mainly attributed to the inconsistency of wound bed evaluation after ED. Furthermore, this is the first study that uses video for remote wound bed evaluation after ED and burn trauma.

## CONCLUSIONS

Although ED users feel confident in its application, IR of wound bed interpretation after application and eventual treatment decision is rather low. However, IR was significantly higher in participants with more experience in ED, thus indicating a learning curve for successful ED utilization. In addition, expert users of ED deemed video analysis as feasible and beneficial as well as useful for education in post-ED wound bed evaluation and decision-making.

## Supplementary Material

iraa218_suppl_Supplementary_Video_2

iraa218_suppl_Supplementary_Video_3

iraa218_suppl_Supplementary_Video_4

iraa218_suppl_Supplementary_Video_5

iraa218_suppl_Supplementary_Video_6

iraa218_suppl_Supplementary_Video_7

iraa218_suppl_Supplementary_Video_8

iraa218_suppl_Supplementary_Video_9

iraa218_suppl_Supplementary_Video_10

iraa218_suppl_Supplementary_Video_11

iraa218_suppl_Supplementary_Video_12

iraa218_suppl_Supplementary_Video_13

iraa218_suppl_Supplementary_Video_14

iraa218_suppl_Supplementary_Video_15

## Notes

This research did not receive any specific grant from funding agencies in the public, commercial, or not-for-profit sectors.
